# Organization and evaluation of generalist palliative care in a Danish hospital

**DOI:** 10.1186/s12904-015-0022-2

**Published:** 2015-05-06

**Authors:** Heidi Bergenholtz, Bibi Hølge-Hazelton, Lene Jarlbaek

**Affiliations:** The Regional Research Unit, Region Zealand, Denmark; Roskilde/Køge Hospitals, Region Zealand, Denmark; The Research Unit for General Practice and Section of General Practice Department of Public Health, University of Copenhagen, Copenhagen, Denmark; PAVI, Knowledge Centre for Rehabilitation and Palliative Care, National Institute of Public Health, University of Southern Denmark, Copenhagen, Denmark

**Keywords:** Palliative care, Generalist palliative care, Hospital, Organization, Evaluation, Accreditation

## Abstract

**Background:**

Hospitals have a responsibility to ensure that palliative care is provided to all patients with life-threatening illnesses. Generalist palliative care should therefore be acknowledged and organized as a part of the clinical tasks. However, little is known about the organization and evaluation of generalist palliative care in hospitals. Therefore the aim of the study was to investigate the organization and evaluation of generalist palliative care in a large regional hospital by comparing results from existing evaluations.

**Methods:**

Results from three different data sets, all aiming to evaluate generalist palliative care, were compared retrospectively. The data-sets derived from; 1. a national accreditation of the hospital, 2. a national survey and 3. an internal self-evaluation performed in the hospital. The data were triangulated to investigate the organization and evaluation of palliative care in order to identify concordances and/or discrepancies.

**Results:**

The triangulation indicated poor validity of the results from existing methods used to evaluate palliative care in hospitals. When the datasets were compared, several discrepancies occurred with regard to the organization and the performance of generalist palliative care. Five types of discrepancies were found in 35 out of 56 sections in the fulfilment of the national accreditation standard for palliative care. Responses from the hospital management and the department managements indicated that generalist palliative care was organized locally – if at all – within the various departments and with no overall structure or policy.

**Conclusions:**

This study demonstrates weaknesses in the existing evaluation methods for generalist palliative care and highlights the lack of an overall policy, organization and goals for the provision of palliative care in the hospital. More research is needed to focus on the organization of palliative care and to establish indicators for high quality palliative care provided by the hospital. The lack of valid indicators, both for the hospital’s and the departments’ provision of palliative care, calls for more qualitative insight in the clinical staff’s daily work including their culture and acceptance of the provision of palliative care.

## Background

During the last decade it has been emphasized that palliative care is relevant for all life-threatening diseases – not only cancer [1]. According to WHO, palliative care need to be a priority across the healthcare sector and must be established through an overall policy to ensure its structure and financing at all levels [1,2]. At the policy level, this seems to be well accepted [3,4]. In several countries, including Denmark, palliative care is organized at two levels: 1. generalist palliative care and 2. specialist palliative care [3,5]. Generalist palliative care is defined as care provided to those affected by life-threatening diseases as an integral part of standard clinical practice by any healthcare professional who is not part of a specialist palliative care team. So, in hospitals, generalist palliative care refers to the care provided by professionals working in non-palliative departments, while specialist palliative care refers to care provided by palliative units [3]. In many countries approximately half of all deaths occur in hospitals [6,7], and in western countries up to 75% of people die from chronic progressive diseases [1]. Hospitals therefore have a significant responsibility to offer and initiate palliative care, and from a quantitative perspective, most palliative care is provided at the generalist level. The implementation of palliative care programmes has been shown to affordably improve the quality of care for patients with palliative needs in healthcare organizations [8]. Despite the increasing focus on generalist palliative care, knowledge concerning its organization, evaluation and quality in hospitals is very sparse. Few studies have addressed the implementation of palliative care programmes in hospitals. In California, a recent study showed an increase in the prevalence of palliative care programmes in hospitals from 17% in 2000 to 44% in 2011 [9]. Other studies have shown considerable variations in palliative care practices in hospitals [10,11].

In Denmark, palliative care is approached in the hospitals’ national accreditation procedure, the ‘Danish Healthcare Quality Programme’ - DDKM (a Danish abbreviation for ’Den Danske Kvalitets Model’) in the Standard 2.19.1: ‘Palliative care of the incurable patient and the patient’s relatives’. This standard aims at securing that the institution provides worthy, respectful, evidence-based palliative treatment to the incurable patient, as well as support and care for the patient’s relatives.

In June 2011 a large regional hospital went through an accreditation procedure. Later in 2011, a nationwide survey on the Danish hospitals’ organization and structure of generalist palliative care was carried out by The Knowledge Centre for Rehabilitation and Palliative Care in Denmark (PAVI-survey), with questionnaires sent to all hospital managements and managers of clinical departments [12]. Two of the questions concerned the departments’ fulfilment of the two indicators for Standard 2.19.1. Furthermore, during the 1^st^ quarter of 2012, the hospital carried out a local evaluation of the palliative care standard. In this study, results from these three different approaches studying the generalist palliative care in hospitals were triangulated. The hospital became the subject for testing the hypothesis that the comparison of independently collected data can provide a more precise and detailed picture of the organization and evaluation of generalist palliative care in hospitals.

### Aim

The aim of this study was to investigate the organization and evaluation of generalist palliative care in the hospital setting using three existing, independently collected data sources in a Danish hospital.

## Methods

This is a retrospective study, where a large regional hospital, which had been the object of three independent evaluations, all describing different aspects of the hospital’s delivery of palliative care, was chosen to test the hypothesis of the study. The conditions at this hospital were considered representative of conditions at the large majority of hospitals in Denmark. Results from the three evaluations were triangulated to describe and identify concordances and/or inconsistencies in the organization, evaluation, prioritization and administration of generalist palliative care.

The hospital was a large, regional teaching hospital with one hospital management and four hospital units (referred to as units 1–4) located in four nearby towns. The hospital covered 30 clinical specialties, had 1,060 beds and 4500 employees. Some of the departments had sections located in more than one unit, though they were led by one departmental management.

Palliative guidelines were developed in April 2011 for common use in all hospitals located in the same region as the hospital in question. The guidelines were based on the requirements described in DDKM’s Standard 2.19.1 and approved by the Head of the Palliative Care Unit in the case hospital. The guidelines were accessible to all staff using the region’s internal document management system.

**Figure 1:**
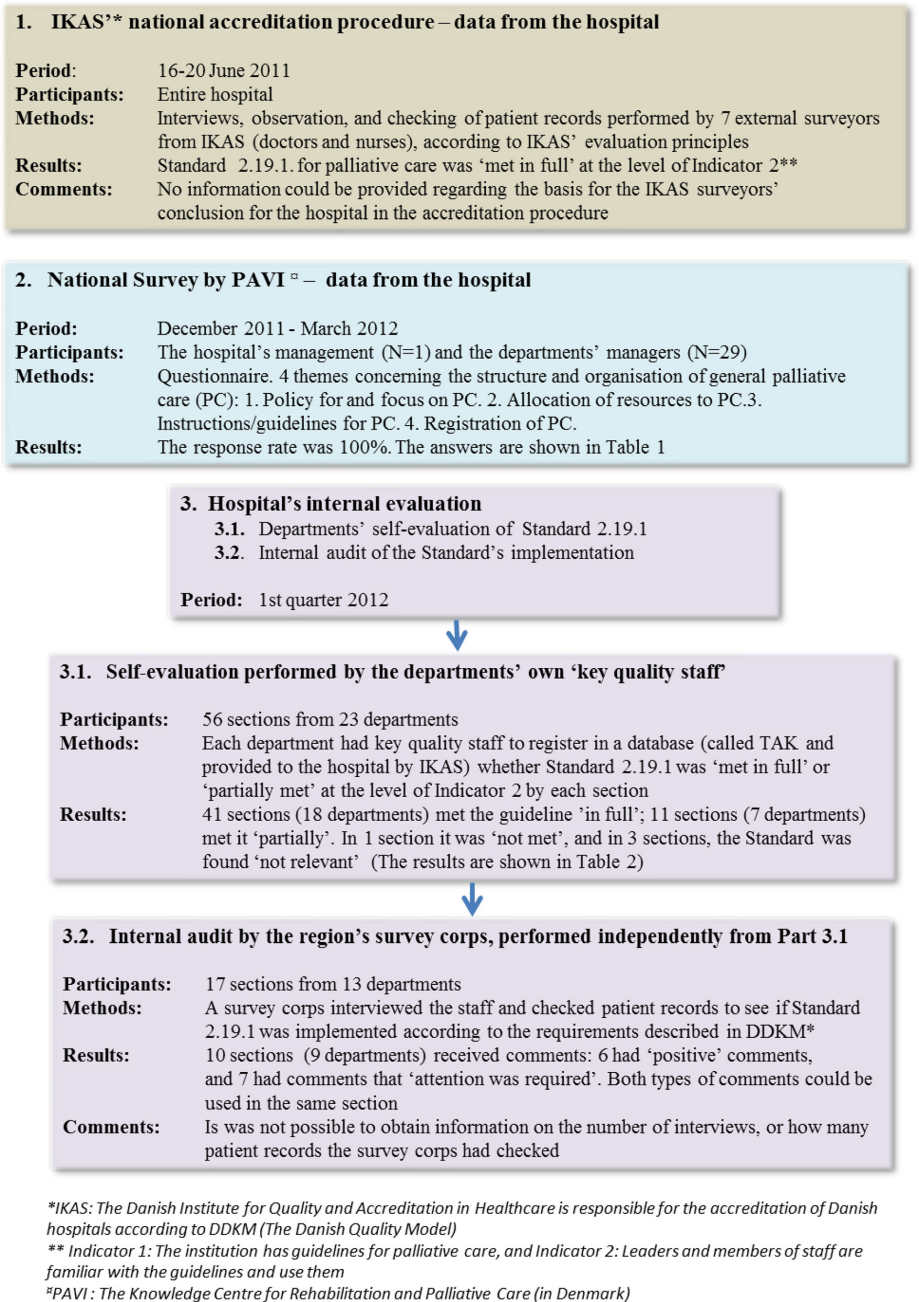
The three datasets used for triangulation.

In the period from summer 2011 to spring 2012, the hospital was the object of three different studies/evaluations, where data on the hospital’s approach to the delivery of generalist palliative care could be acquired (Figure 1). All three studies had evaluated Standard 2.19.1 for palliative care in different ways, and the organization and administration of generalist palliative care was approached more thoroughly in the PAVI survey. Results from the three individual studies will be presented in the methods section, because they comprise the data on which the triangulation is based. The presentation of these results in the methods section will provide the basis for interpreting the results of the triangulation and the subsequent discussion.

### The external accreditation procedure by IKAS

The hospital initially went through the national accreditation procedure performed by The Danish Institute for Quality and Accreditation in Healthcare, known as IKAS, which manages, develops and plans the DDKM programme [13]. IKAS uses accreditation standards to ensure an impartial assessment of the hospitals’ conditions for providing services of high quality. As mentioned earlier palliative care is approached in DDKM as Standard 2.19.1; ‘Palliative care of the incurable patient and the patient’s relatives’. The aim of this standard is to ensure that the patient experiences worthy, respectful, empathic palliative treatment when active treatment is pointless and that the patient’s relatives are involved in the palliative course in a worthy and respectful way, when desired by the patient. The target group for this standard is managers and staff in sections providing palliative treatment. Two indicators have to be fulfilled to comply with the standard – Indicator 1: presence of guidelines for the institution’s provision of palliative care and Indicator 2: managers and staff are familiar with and use the guidelines.

#### Results from IKAS’ data

IKAS evaluated Standard 2.19.1 as having been ‘met in full’ by the hospital at the level of Indicator 2. Because of the existence of common palliative guidelines for all hospitals in the region, it is evident that the standard could be evaluated as fulfilled at the level of Indicator 1: the presence of guidelines. However, on the level of Indicator 2 it was not possible to obtain a specification from IKAS regarding the basis for the surveyors’ conclusion. IKAS’ general evaluation principles can be found on the internet [13], but these principles do not offer any further clarification on the subject.

### The PAVI-survey

The hospital participated in the nationwide PAVI-survey, designed to map the organization and structure of Danish hospitals’ provision of generalist palliative care. All clinical departments with patient contact received a questionnaire concerning the organization and structure of palliative care in the department, categorized into different themes. The national overall response rate among 410 departments was 78%, and data from the case hospital were extracted for this study. A report of the full survey can be found elsewhere [12].

#### Results from PAVI-survey

Both the management of the hospital (n = 1), and all of the hospital’s clinical department managers (n = 29) responded to the survey. One manager from a surgical department declined to have palliative patients among the department’s clientele and commented that the department ‘did not treat cancer patients’. Six departments answered ‘no’ to provide palliative care – one paediatric, one surgical, and four miscellaneous. The remaining 22 departments’ managers confirmed that they sometimes did provide palliative care, and they were further questioned with regard to their departments’ organization and provision of palliative care. The responses relevant for this study are shown in Table 1, and compared with answers from the hospital’s management.

**Table 1:** **The hospital’s and departments’ managers’ responses to the PAVI-survey concerning organization and prioritisation of palliative care (PC)**

**Hospital’s management (N = 1)**		**Departments’ managers (N = 22)**	**Disagreements**
**Question answer**		**Question answer**	**Between hospital’s and departments’ managers**
Theme 1. Policy for and focus on palliative care			
Does the hospital have a general policy for PC*?	Does the hospital have a general policy for PC?		
	Yes:	6 (27%)	Disagreement
	No:	11 (50%)	
	Don’t know	5 (23%)	
	Does the department have a policy for PC?		
	Yes:	9 (41%)	Incomparable
	No:	12 (55%)	
	Don’t know:	1 (4%)	
	Does the department focus on PC?		
	Yes:	22 (100%)	Incomparable
	Who creates focus on PC in the department?		
	Department’s managers:	18 (82%)	
	Hospital’s management:	6 (27%)	
	Doctors:	15 (64%)	Incomparable
	Caregivers:	17 (77%)	
	Dedicated staff:	2 (9%)	
	Specialised PC:	9 (41%)	
	Other:	1 (4%)	
Are there an ongoing dialogue between the hospital’s and departments’ managers	Does the department management have an ongoing dialogue with the hospital’s management concerning PC?		
	Yes, satisfactory:	6 (27%)	Disagreement
	Yes, but insufficient:	1 (4%)	
	No, dialogue is missing:	5 (23%)	
	No, not applicable:	3 (14%)	
	No, not necessary:	7 (32%)	
Theme 2: Allocation of resources to palliative care			
The hospital’s framework for PC involves?	Has the hospital’s management provided a framework for the department’s PC?		
	Yes:	6 (27%)	Disagreement
Time provided to increase staff’s qualifications	No:	13 (59%)	
	No answer:	3 (14%)	
	Has the department allocated resources specifically for PC?		
	Yes:	7 (32%)	Incomparable
	No:	15 (68%)	
	Theme 3. Instructions/guidelines for palliative care		
Has the hospital established general instructions/guidelines for PC?	Does the department have instructions/guidelines for PC?		
			Disagreement
Don’t know	Yes:	12 (55%)	
	No:	10 (45%)	
Has the hospital a procedure for implementation of general instructions and guidelines?			
Yes			Incomparable
Theme 4: Registration of palliative care			
Is PC registered in the department?			
	Yes:	7 (32%)	Incomparable
	No:	9 (41%)	
	Don’t know:	3 (14%)	
	N/A:	3 (14%)	
Does the hospital have administrative tools for the registration of general PC?	Does the department have administrative procedures for the registration of PC?		
	Yes:	4 (18%)	Disagreement
	No:	13 (59%)	
Don’t know:	Don’t know:	4 (18%)	
	N/A:	4 (4%)	
Does the hospital lack administrative tools for general PC?	Is there a need for administrative procedures for the registration of PC?		
	Yes:	1 (4%)	Disagreement
	No:	6 (27%)	
Don’t know:	Don’t know:	6 (27%)	
	Did not answer::	9 (41%)	
	Are there DRG-codes for the registration of the department's PC?		
	Yes:	5 (23%)	Incomparable
	No:	7 (32%)	
	Don’t know:	10 (45%)	

### The hospital’s internal evaluation

The hospital went through a self-evaluation as part of the accreditation process. IKAS recommends that self-evaluations be carried out between the external accreditation procedures, which take place every three years. The aim of self-evaluations is to ensure and encourage development and fulfilment of the standards. The self-evaluation consists of two parts – 3.1: a self-evaluation performed by key quality personnel in the clinical departments, and 3.2: an audit conducted by a survey corps in selected departments (Figure 1). Key quality personnel are persons, usually nurses, employed in the departments with a view to implementing the accreditation-standards. Survey corps are trained in the regions where the hospitals are located. The internal survey aims to evaluate to what extent the palliative guideline has been implemented and to identify needs for improvement in order to fulfil the indicators [13].

#### Results from the hospital’s internal evaluation

##### Part 1) The departments’ self-evaluation

Among 23 departments, 56 evaluations were retrieved from the different wards and outpatient clinics (Table 2). Six of the hospital’s 29 clinical departments did not participate in the self-evaluation – two medical departments and four miscellaneous. The results were registered by the key quality staff in a documentation system called TAK, provided to the hospital by IKAS. Of the 56 evaluations, 41 (representing 18 departments) were evaluated as having met the indicator targets in full (Table 2).

**Table 2:** **Departments participating in the PAVI-survey and in the hospital’s internal evaluation of Standard 2.19.1**

PAVI-survey*			Hospital’s self evaluation**							
			**Departments’ self-evaluation**						**Audit**	
			**Sections involved**	**[Departments involved]**	**Standard 2.19.1 was:**				**Sections involved**	**Comments**
**Type of department**	**Responders**	**Providers of palliative care**			**Fully met**	**Partially met**	**Not met**	**Not relevant**		
N	29	22	56	[23]	41 [18]	11 [7]	1 [1]	3 [3]	17 [13]	10 [9]
Medical	8	8	16	[6]	9 [3]	6 [4]	1	0	6 [4]	3 [2]
Surgical	7	5	19	[7]	15 [6]	4 [2]	0	0	5 [3]	3 [3]
Paediatric	2	1	6	[2]	5 [2]	0	0	1	0	0
Oncology/haematology	2	2	2	[2]	2 [2]	0	0	0	2 [2]	2 [2]
Anaesthesiology	3	3	8	[3]	7 [3]	1	0	0	1	1
Gynecology/obstetrics	2	2	4	[2]	3 [2]	0	0	1	2	1
Miscellaneous	5	1	1	[1]	0	0	0	1	1	0

*A national survey concerning the organisation and structure of palliative care in Danish clinical hospital departments (N = 410). Here, the responses from the case hospital’s 29 participating departments are shown.** The self evaluation concerned 56 sections’ fulfilment of Standard 2.19.1: ‘Treatment of the incurably ill patient and care for the patient’s relatives’, Version 1, at the level of Indicator 1: The institution has guidelines for palliative care, and Indicator 2: Leaders and members of the staff are familiar with the guidelines and use them.# Of the 29 PAVI-survey responders, 6 departments did not join the selfevaluation: 2 medical departments, 1 audiology, 1 eye, 2 emergency room.

¤Miscellaneous consists of departments: Eye department, dermatology, audiology and 2 ER’s (emergency room).

§All numbers written in parenthesis refers to the number of departments (in all 23 departments encompassing the 56 sections involved in the self-evaluation).

An overview of the departments that participated in the hospital’s self-evaluation and in the PAVI-survey is shown in Table 2.

##### Part 2) Audit of selected departments

The survey corps, trained by the region, had planned to visit all the somatic departments, but only 13 departments were audited (the reasons for this could not be revealed). The departments’ managers were contacted prior to the visit and they agreed with the surveyors on which sections should be audited. The audit was carried out in 17 sections from the 13 departments. Ten departments received comments on the palliative standard 2.19.1 (Table 2). The findings could be categorized as a “positive grade” (fulfilling the indicator targets) or findings that “required attention” (deviation from fulfilling the indicator targets), and it was possible to get both types of comments within the same section. Six sections did not receive any comments, six sections were graded ‘positive’ and seven sections had findings that ‘required attention’. The comment ‘require attention’ could, for instance, refer to a lack of knowledge on the palliative standard, a lack of private rooms for conversations, a lack of documentation about information given to relatives, or non-adherence to the Edmonton Symptom Assessment System (ESAS) as requested in the hospital’s common palliative guideline.

### Statistics

Descriptive statistics are used to present the results of the triangulation.

### Ethical considerations

The study was performed in accordance with the Declaration of Helsinki. The Danish Data Protection Agency registered the study. According to the Regional Committee for Medical research, ethical approval was not required. Consent to use the data was obtained from the hospital’s management and the departments’ managers.

## Results

The results from the triangulation of the three studies will be presented in this section.

The departments that participated in the PAVI-survey and the internal evaluation of Standard 2.19.1 are shown in Table 2.

### The hospital’s fulfilment of Standard 2.19.1

Five types of discrepancies could be identified when the results from the three studies of the hospital’s and its departments’ fulfilment of Standard 2.19.1 were triangulated. The discrepancies could be identified among 35 sections, representing 19 departments, and the distribution of these discrepancies is shown in Table 3:

**Table 3:** **Discrepancies in the departments/sections evaluations of Standard 2.19.1 were identified in 35 cases**

**Sections**	**Hospital’s self-evaluation**		**PAVI -survey**		**IKAS-data**	**Discrepancies**
**N = 35**	**Departments’ self evaluationStandard met?**	Internal audit*	**Providing palliative care**	Instructions/guidelines for palliative care**	**Accreditation of standard 2.19.1**	**1-5**
Unit 1 Medical	Fully	NC	Yes	No		1
Unit 1 Medical	Fully	NA	Yes	No		1
Unit 1 Medical	Fully	NA	Yes	No		1
Unit 1 Medical	Fully	NA	Yes	No		1
Unit 1 Medical	Fully	NA	Yes	No		1
Unit 1 Medical	Fully	NA	Yes	No		1
Unit 1 Medical	Partially	RA	Yes	No		1 + 5
Unit 1 Medical	Partially	NA	Yes	Yes		5
Unit 2 Medical	Partially	NA	Yes	Yes		5
Unit 2 Medical	Not met	NC	Yes	Yes		5
Unit 2 Medical	Partially		Yes	Yes		5
Unit 2 Medical	Partially		Yes	Yes		5
Unit 3 Medical	Partially	RA + P	Yes	Yes		5
Unit 1 Oncology	Fully	RA	Yes	Yes	Indicator 1 and Indicator 2 were both met in full by all the departments	4
Unit 1 Haematol	Fully	RA	Yes	No		1 + 4
Unit 2 Surgical	Fully	NA	No	NQ		3
Unit 2 Surgical	Fully	NA	No	NQ		3
Unit 2 Surgical	Fully	NA	No	NQ		3
Unit 2 Surgical	Fully	NA	No	NQ		3
Unit 2 Surgical	Partially	P	Yes	Yes		5
Unit 2 Surgical	Partially	NA	Yes	Yes		5
Unit 1 Surgical	Fully	NA	Yes	No		1
Unit 3 Surgical	Partially	RA	Yes	No		1 + 5
Unit 3 Surgical	Partially	P	Yes	No		1 + 5
Unit 3 Surgical#	Fully	NA	No pall ptts #	NQ		3
Unit 3 Gyn/obs	Fully	RA + P	Yes	Yes		4
Unit 1 Gyn/obs	Not Relevant	NA	Yes	Yes		2
Unit 2 Anesth	Partially	NA	Yes	No		1 + 5
Unit 2 Anesth	Fully	RA	Yes	No		1 + 4
Unit 1 Miscellaneous	Not relevant	NC	Yes	No		2
Unit 1 Paediatric	Fully	NA	No	NQ		3
Unit 1 Paediatric	Fully	NA	No	NQ		3
Unit 1 Paediatric	Fully	NA	No	NQ		3
Unit 3 Paediatric	Fully	NA	Yes	No		1
Unit 3 Paediatric	Fully	NA	Yes	No		1

*Audit’s comments: P - positive - confirm indicator targets were met. RA - requires attention - discrepancy in fulfilling the indicators. NC - no comments. NA - no audit. ** NQ - not questioned survey# according to the PAVI-survey, this department did not treat palliative patients.

¤Types of discrepancies identified in the triangulation of the 3 dataset: Discrepancy 1: Departments stating in the PAVI-survey ‘not to have a palliative guideline/instruction’, while Standard 2.19.1 was fully or partially fulfilled according to the self evaluation.Discrepancy 2: Departments stating in the self-evaluation that use of the standard was “not relevant”, while responding in the PAVI-survey that their department ‘did provide palliative care’. Discrepancy 3: Departments stating in the PAVI-survey ‘not to provide palliative care’, while they fulfilled the standard according to the selfevaluation.Discrepancy 4: Departments that stated to ‘meet the standard in full’ in the self-evaluation, but received a comment in the audit that they ‘required attention’ Discrepancy 5: Departments that stated to fulfil Standard 2.19.1 only “partially” or “not met” in the self-evaluation, but were assessed by IKAS to “meet the Standard in full”, at the level of Indicator 2.

- ***Discrepancy 1:*** In the PAVI-survey 15 departments stated that they did not have a palliative guideline/instruction, while in the self-evaluation they claimed to have fulfilled Standard 2.19.1 fully or partially.
- ***Discrepancy 2:*** Two departments assessed the use of the standard in the self-evaluation as “not relevant” despite having responded in the PAVI-survey that their department provided palliative care.
- ***Discrepancy 3:*** Eight departments did not provide palliative care according to the PAVI-survey, however in the self-evaluation the departments’ internal surveyors all stated that the standard was met in full.
- ***Discrepancy 4:*** In the internal audit, four of the sections had comments that ‘required attention’, despite having responded in the self-evaluation that they met the standard in full.
- ***Discrepancy 5:*** In the self-evaluation, 12 departments assessed Standard 2.19.1 as having been “partially” met or “not met”, despite the fact that Standard 2.19.1 was assessed by IKAS as having been “met in full” at the level of Indicator 2.

For six departments, more than one discrepancy could be identified.

### Organization of generalist palliative care (PAVI-survey)

The responses from the hospital’s management and the departments’ managers in the PAVI-survey were compared. Several disagreements were identified concerning the four themes shown in Table 1: 1. Policy for and focus on palliative care, 2. Allocation of resources to palliative care, 3. Instructions/guidelines for palliative care, and 4. Registration of palliative care. The hospital’s management answered ‘no’ to the survey’s first question, “Does the hospital have a general policy for palliative care?”, while 27% of the departments’ managers answered ‘yes’ to the same question. From the responses by the departments’ managers, 41% responded that their own department had a policy for palliative care. The hospital’s management did not find itself engaged in a dialogue with the departments concerning palliative care; however, 27% of the departments’ managers found the dialogue satisfactory, 23% missed a dialogue, and 32% found it ‘unnecessary’. The hospital’s management was unaware of the guideline for palliative care common to all hospitals in the region. Despite the existence of this common guideline, almost half of the departments’ managers (45%) responded that their department had no guidelines for the provision of palliative care (as requested to fulfil Indicator 2 of the Standard 2.19.1). The focus on palliative care was primarily created by the department managers, doctors and caregivers.

The hospital’s management responded to provide resources for generalist palliative care, by allowing the staff time to improve their qualifications. However, only seven out of the 22 departments (32%) responded that resources had been allocated specifically for the departments’ provision of generalist palliative care. Neither the hospital’s management nor the departments’ managers were concerned that the departments had no registration procedures for their provision of palliative care. The hospital’s management was unaware whether it was at all possible to register generalist palliative care, and only one department expressed a need for this type of registration.

## Discussion

This study has shown that generalist palliative care was organized and prioritized differently within the various departments, and that there was no overall policy or goal for the hospital’s provision of palliative care. The Danish National Board of Health’s recommendations for palliative care include having a common policy for palliative care in place, as well as joint efforts from the hospitals to identify patients in need of palliative care [3], although appropriate methods for early identification of these patients remains to be documented [14]. Evidence exists that early, targeted and systematic palliative care for patients with life-threatening diseases allows for better symptom management and fewer hospitalisations [15-17]. To support this, integrated trajectory palliative care models [18] have been recommended and developed in order to ensure that such services are well coordinated. However, knowledge regarding integration of these models in generalist palliative care is yet to be explored. In general, systemically implemented clinical pathways have indicated a beneficial effect, with fewer hospital complications and improved documentation [19]. However, these effects have not yet been shown specifically with regard to end-of-life care pathways [20].

### The hospital’s fulfilment of the accreditation standard for palliative care

The triangulation of the three datasets identified various discrepancies when the responses to similar questions concerning the fulfilment of the standard for palliative care were compared. The national accreditation procedure concluded that the hospital completely fulfilled the quality standard for palliative care. This conclusion must be designated as invalid, considering the discrepancies that were identified when the results from the two other datasets were evaluated. To fulfil the quality standard it was mandatory to have guidelines in place for the departments’ provision of palliative care, and to know about and use the guidelines. However, in the PAVI survey 15 out of 22 department managers declined to have instructions for palliative care, and in the self-evaluation the standard was not met or only partially met by several departments. This raises the question on ‘how to evaluate the quality of palliative care’ – which indicators and which methods are useful and valid? Based on this study, it seems apparent that the quality of palliative care provided by the hospitals cannot be described or certified only by performing the national accreditation procedure. Even if the results of the DDKM accreditation had seemed valid, there is still a lack of proof between the existence and awareness of guidelines for palliative care and the provision of high quality palliative care.

There is an ongoing discussion regarding to what extent the use of ‘health sector accreditation’ improves the quality of care provided to patients and their relatives [21]. In a Cochrane Review from 2011 [22], the external inspection of compliance with other kinds of standards could not be demonstrated to improve quality; however, only a small number of studies have focused on this issue. In the US, the Joint Commission on Accreditation of Healthcare Organizations, certification of palliative care programs, has been included in the accreditation procedures for several years [23]. Hospitals accredited by The Joint Commission have shown a higher performance with regard to adherence to quality measures compared with non-accredited hospitals [24]. Whether this adherence results in better quality, or whether the results were due to biases caused by differences in the hospitals’ characteristics, remains unclear. So, internationally the discussion of accreditation procedures as usable markers for good quality continues [25]. Detailed quality indicators for palliative care have been lacking [26], and efforts are being made to develop quality indicators applicable to all palliative care settings [27]. Recently, palliative care experts were asked to score the relevance and usability of different quality indicators for national healthcare systems’ organization of palliative care for patients with cancer and dementia [28]. They seemed to have reached consensus on 23 indicators covering both the access to a specialist palliative care team, infrastructure, continuity, documentation and education of all professionals providing palliative care. However, the difficulties in measuring the quality of palliative care can advocate for more qualitative studies [29]. The patients’ and the relatives’ viewpoints must be considered [30], as well as the challenges the professionals are facing in caring for patients in common clinical departments [31-34].

### Organization of generalist palliative care (disagreements between hospital and departmental managements)

In our study, the delivery of high quality palliative care did not seem to be regarded as a shared task for the hospital’s departments, judged by the disagreements identified in the answers from the hospital’s management and the departments’ managers. Even within single departments, the triangulation of the data revealed discrepancies between the responses from the departments’ managers and their key quality staff, and between the key quality staff and the region’s audit corps. So, both on the level of the hospital and among several of the departments, there was no common approach to ensure a high quality palliative care at the generalist level for patients and their relatives.

The PAVI-survey revealed a general lack of resources specifically allocated to the provision of generalist palliative care. It remains speculative whether this means that this type of care is not regarded as a part of the department’s productivity and ‘just’ has to be delivered within the ‘existing frames’ of the department’s activities and budget. The managers did not seem specifically interested in registering the departments’ provision of palliative care. In fact, within the hospitals’ reimbursement system no DRG-codes exist for generalist palliative care; only on the level of specialized palliative care is there a DRG-code available for palliative treatment. It remains unknown whether the lack of productivity registration influences the quality of the care delivered. Research has shown that financial support in health care may have an effect in changing the practice of healthcare professionals when a payment is given for each service, but without evidence in patient outcome [35]. Factors such as lack of resources and lack of time have been shown to be barriers to providing palliative care in the hospital setting [36]. Time is necessary to get to “know” the patient to be able to offer individualized care and relief [37]. If no extra resources are allocated to this aspect of caring for patients with life-threatening diseases, then it is likely that the time-consuming tasks will be given lower priority when the productivity of the departments is in focus.

In very many ways the organization and accreditation of the Danish hospital system resembles the hospital and accreditation systems in other western countries [38]. Therefore, the implications and relevance of the results presented here are likely to be of importance both to healthcare professionals and continuous research in the provision of generalist palliative care, and the search for valid quality indicators. Hospital systems must relate to their reputation of being ‘the most undesirable setting for place of death’ [39] and to the fact that the quality of end-of-life care for their dying patients is described worldwide as poor [40]. The challenges of organizing and evaluating generalist palliative care are issues for the Danish healthcare system as well as for European systems and globally.

### Strengths and limitations

The use of existing data sources in data triangulation is both a weakness and a strength. It is a weakness because we did not know the considerations that lay behind the responses, and owing to the retrospective design we were unable to influence the content of the questions for our purpose and the persons questioned. Two of the three evaluations of the ‘palliative care performance’ were based on self-reporting. A tendency for over-estimation of adherence to guidelines in self-reported situations has been demonstrated [41]. This advocates that self-reporting should not be the only method used to evaluate clinical practice. The strength of the data triangulation lies in the independence between the three data sets, which allowed us to approach biases caused by self-reported over-estimation, and to question the validity of the external accreditation procedure. Furthermore, the variation of data can describe the field with more breadth.

This study only encompasses results from a single hospital, which is a limitation. However, the hospital was a large regional hospital, with an organization and administration similar to other hospitals of this size in Denmark. When the responses from the hospital were compared with the responses reported in the national PAVI-study [12], the hospital also appeared to be representative of hospitals in Denmark in general.

## Conclusion

This study demonstrates a lack of an overall policy, organization and goals for the provision of palliative care, as recommended by the national recommendations for palliative care. Invalid results and weaknesses in the existing evaluation methods for the quality of generalist palliative care have been demonstrated. There is a need for more research focusing both on optimization of the hospitals’ and their clinical departments’ organization of generalist palliative care, and for indicators that can ensure that patients and relatives receive high quality palliative care when they need it. The lack of valid indicators both for the hospitals’ and the departments’ organization and provision of palliative care, and the quality of the care, calls for more qualitative insight into the clinical staff’s daily work, their culture and acceptance of the provision of palliative care to those in need. So far, no gold standard for high quality generalist palliative care seems to exist.

## Abbreviations

DDKM: The Danish Healthcare Quality Programme
IKAS: Institute for Quality and Accreditation in the Danish National Health Service
PAVI: Knowledge Centre for Rehabilitation and Palliative Care (in Denmark)
DRG: Diagnosis-related group
